# Supplementary material for: Accumulation of F-actin drives brain aging and limits healthspan in Drosophila
Source: Nat Commun. 2024 Oct 25;15:9238. doi: 10.1038/s41467-024-53389-w (PMC11512044; doi:10.1038/s41467-024-53389-w)
Supplement: Supplementary file 1 — Supplementary Information [file 41467_2024_53389_MOESM1_ESM.pdf]

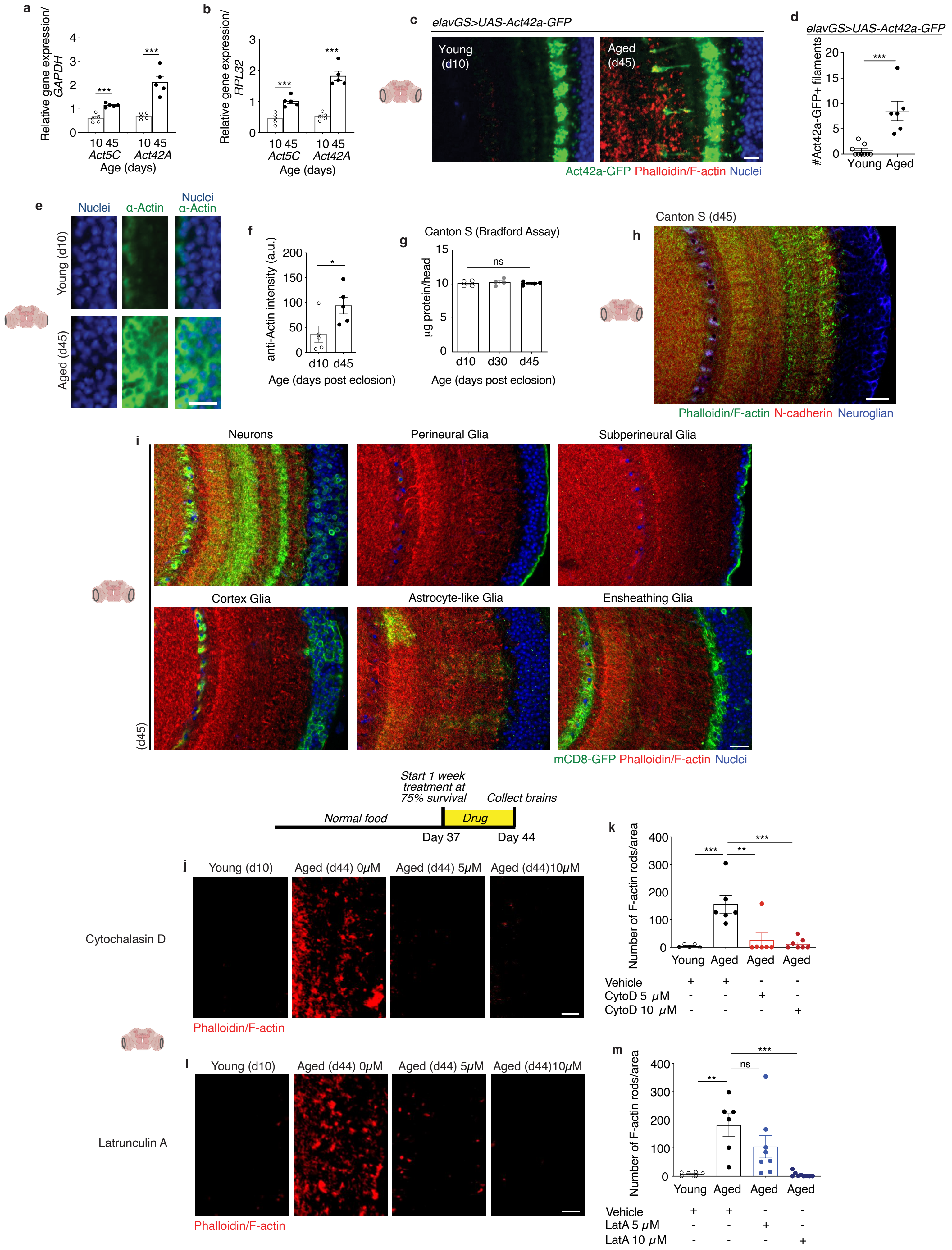

a
Relative gene expression/ GAPDH
10 45 Act5C 10 45 Act42A
Age (days)
b
Relative gene expression/ RPL32
10 45 Act5C 10 45 Act42A
Age (days)
c
elavGS>UAS-Act42a-GFP
Young (d10)
Aged (d45)
Act42a-GFP Phalloidin/F-actin Nuclei
d
elavGS>UAS-Act42a-GFP
#Act42a-GFP+ filaments
Young Aged
e
Nuclei α-Actin Nuclei α-Actin
Young (d10)
Aged (d45)
f
anti-Actin intensity (a.u.)
d10 d45
Age (days post eclosion)
g
Canton S (Bradford Assay)
µg protein/head
ns
d10 d30 d45
Age (days post eclosion)
h
Canton S (d45)
Phalloidin/F-actin N-cadherin Neuroglian
i
Neurons
Perineural Glia
Subperineural Glia
Cortex Glia
Astrocyte-like Glia
Ensheathing Glia
(d45)
mCD8-GFP Phalloidin/F-actin Nuclei
Start 1 week treatment at 75% survival
Collect brains
Normal food
Drug
Day 37
Day 44
j
Young (d10) Aged (d44) 0µM Aged (d44) 5µM Aged (d44)10µM
Cytochalasin D
Phalloidin/F-actin
k
Number of F-actin rods/area
Young Aged Aged Aged
Vehicle + + - -
CytoD 5 µM - - + -
CytoD 10 µM - - - +
l
Young (d10) Aged (d44) 0µM Aged (d44) 5µM Aged (d44)10µM
Latrunculin A
Phalloidin/F-actin
m
Number of F-actin rods/area
ns
Young Aged Aged Aged
Vehicle + + - -
LatA 5 µM - - + -
LatA 10 µM - - - +

Supplementary Figure 1. Actin polymerization increases in aging *Drosophila* neurons and can be disrupted pharmacologically.

(a) qPCR analysis of *Act5c* and *Act42a* relative to *GAPDH* in fly heads on days 10 and 45 post eclosion from Canton S flies. n = 5 biological replicates with 5 dissected heads pooled per replicate. ***p (*Act5c*) = 0.0002; ***p (*Act42a*) = 0.0004, unpaired t-tests.

(b) qPCR analysis of *Act5c* and *Act42a* relative to *RPL32* in fly heads on days 10 and 45 post eclosion from Canton S flies. n = 5 biological replicates with 5 dissected heads pooled per replicate. ***p (*Act5c*) = 0.0009; ***p (*Act42a*) < 0.0001, unpaired t-tests.

(c) Immunostaining of brains at 63x magnification from young (10-day-old) and aged (45-day-old) *elavGS>UAS-Act42a-GFP* flies, showing F-actin (red channel, phalloidin), Act42a-GFP (green channel), and nuclear DNA (blue channel, To-Pro-3). Scale bar is 10 μm.

(d) Quantification of Act42a-GFP+ filaments observed in brain areas as shown in (f). n = 6-9 flies per condition, as indicated. ***p = 0.0006, unpaired t-test.

(e) Immunostaining of brains at 63x magnification from young (10-day-old) and aged (45-day-old) Canton S flies, showing anti-actin (green channel) and nuclear DNA (blue channel, To-Pro-3). Scale bar is 5 μm.

(f) Quantification of actin intensity by anti-actin antibody in brains as shown in (c). n = 5 flies per condition. *p = 0.0399, unpaired t-test.

(g) Quantification of protein concentration by Bradford assay from pools of 5 head homogenates, as in Figure 1g. ns = non-significant, one-way ANOVA, Tukey's multiple comparisons test.

(h) Immunostaining of brains at 63x magnification from aged (45-day-old) Canton S flies, showing F-actin (green channel, phalloidin), anti-neuropil (red channel), and anti-neuroglian (blue channel). Scale bar is 10 μm.

(i) Immunostaining of brains at 63x magnification from aged (45-day-old) flies, showing mCD8-GFP (green channel, phalloidin), F-actin (red channel), and nuclear DNA (blue channel, DAPI). Scale bar is 10 µm. Reporter lines crossed with UAS-mCD8GFP are: *elav-Gal4* (neurons), *NP6293-Gal4* (perineural glia), *Mdr65-Gal4* (subperineural glia), *GMR54H02-Gal4* (cortex glia), *alrm-Gal4* (astrocyte-like glia), and *rumpel-Gal4* (ensheathing glia). Scale bar is 10 µm.

(j) Immunostaining of brains at 63x magnification from young (10-day-old) and aged (44-day-old) Canton S flies given vehicle (DMSO), 5µM cytochalasin D, or 10µM cytochalasin D as indicated from days 37-44 post eclosion, showing F-actin-rich rods in brain optic lobes (red channel, phalloidin). Scale bar is 5 µm. Accompanying diagram indicates drug feeding paradigm.

(k) Quantification F- actin-rich rods by phalloidin stain per 1 mm^2 area of brains as shown in (j). n = 5-7 flies per condition, as indicated. **p = 0.0018, ***p (young vs. aged + vehicle) = 0.0006, ***p (aged + vehicle vs. aged + 10 µM cytochalasin D) = 0.0004; one-way ANOVA, Tukey's multiple comparisons test.

(l) Immunostaining of brains from young (10-day-old) and aged (44-day-old) Canton S flies given vehicle (DMSO), 5µM Latrunculin A, or 10µM Latrunculin A as indicated from days 37-44 post eclosion, showing F-actin accumulation (red channel, phalloidin). Scale bar is 5 µm.

(m) Quantification of the number of F-actin-rich rods by phalloidin stain per 1 mm^2 area of brains as shown in (l). n = 6-9 flies per condition, as indicated. ns = non-significant, **p = 0.0022, ***p = 0.0007; one-way ANOVA, Tukey's multiple comparisons test. Data are presented as scatter plots overlaying mean values +/- SEM.

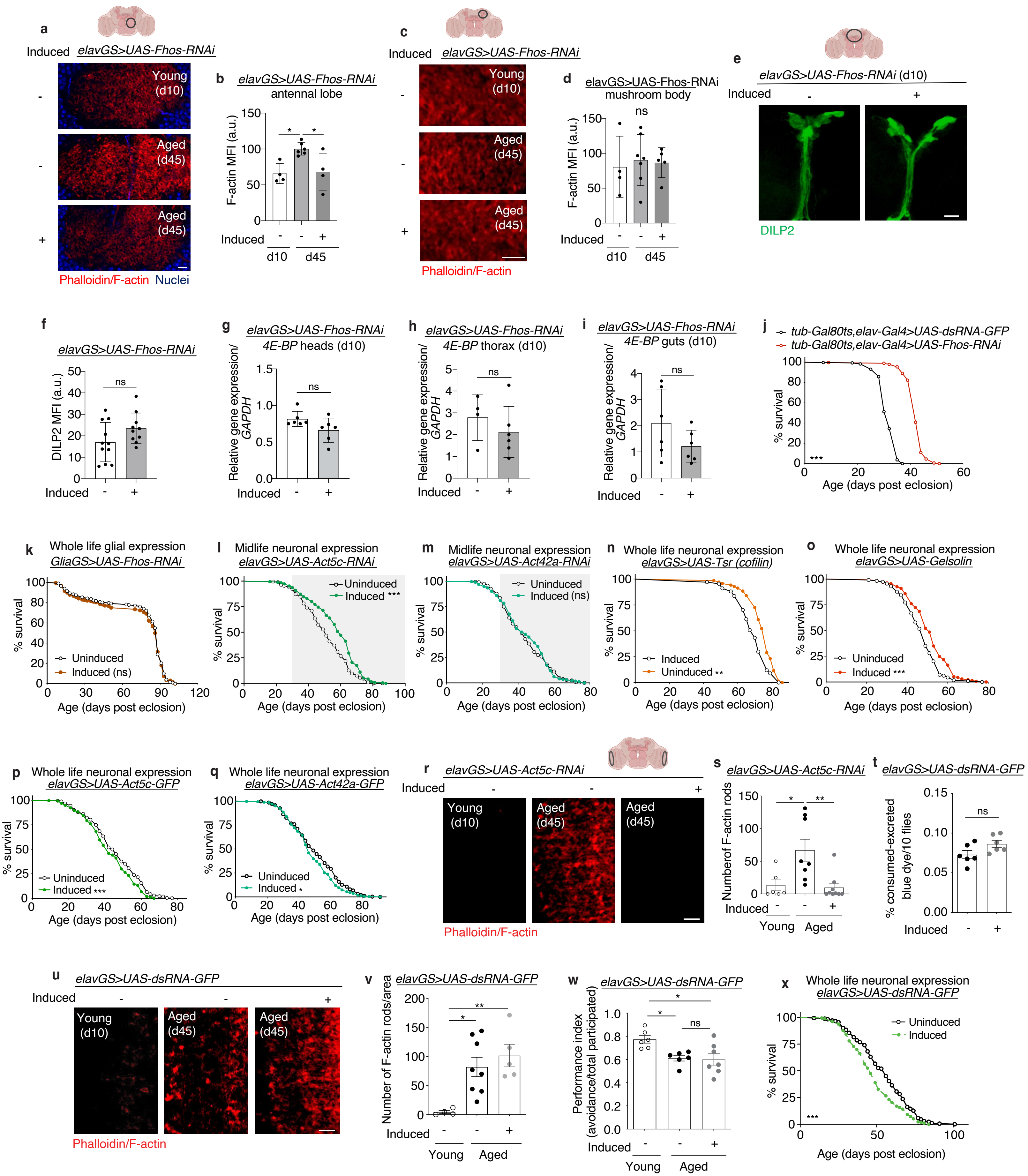

a
Induced elavGS>UAS-Fhos-RNAi
Young (d10)
Aged (d45)
Aged (d45)
Phalloidin/F-actin Nuclei
b
elavGS>UAS-Fhos-RNAi antennal lobe
F-actin MFI (a.u.)
Induced
d10
d45
c
Induced elavGS>UAS-Fhos-RNAi
Young (d10)
Aged (d45)
Aged (d45)
Phalloidin/F-actin
d
elavGS>UAS-Fhos-RNAi mushroom body
F-actin MFI (a.u.)
Induced
d10
d45
e
elavGS>UAS-Fhos-RNAi (d10)
Induced
DILP2
f
elavGS>UAS-Fhos-RNAi
DILP2 MFI (a.u.)
Induced
g
elavGS>UAS-Fhos-RNAi
4E-BP heads (d10)
Relative gene expression/ GAPDH
Induced
h
elavGS>UAS-Fhos-RNAi
4E-BP thorax (d10)
Relative gene expression/ GAPDH
Induced
i
elavGS>UAS-Fhos-RNAi
4E-BP guts (d10)
Relative gene expression/ GAPDH
Induced
j
tub-Gal80ts,elav-Gal4>UAS-dsRNA-GFP
tub-Gal80ts,elav-Gal4>UAS-Fhos-RNAi
% survival
Age (days post eclosion)
k
Whole life glial expression
GliaGS>UAS-Fhos-RNAi
Uninduced
Induced (ns)
% survival
Age (days post eclosion)
l
Midlife neuronal expression
elavGS>UAS-Act5c-RNAi
Uninduced
Induced ***
% survival
Age (days post eclosion)
m
Midlife neuronal expression
elavGS>UAS-Act42a-RNAi
Uninduced
Induced (ns)
% survival
Age (days post eclosion)
n
Whole life neuronal expression
elavGS>UAS-Tsr (cofilin)
Induced
Uninduced **
% survival
Age (days post eclosion)
o
Whole life neuronal expression
elavGS>UAS-Gelsolin
Uninduced
Induced ***
% survival
Age (days post eclosion)
p
Whole life neuronal expression
elavGS>UAS-Act5c-GFP
Uninduced
Induced ***
% survival
Age (days post eclosion)
q
Whole life neuronal expression
elavGS>UAS-Act42a-GFP
Uninduced
Induced *
% survival
Age (days post eclosion)
r
elavGS>UAS-Act5c-RNAi
Induced
Young (d10)
Aged (d45)
Aged (d45)
Phalloidin/F-actin
s
elavGS>UAS-Act5c-RNAi
Numberof F-actin rods
Induced
Young
Aged
t
elavGS>UAS-dsRNA-GFP
% consumed-excreted blue dye/10 flies
Induced
u
elavGS>UAS-dsRNA-GFP
Induced
Young (d10)
Aged (d45)
Aged (d45)
Phalloidin/F-actin
v
elavGS>UAS-dsRNA-GFP
Number of F-actin rods/area
Induced
Young
Aged
w
elavGS>UAS-dsRNA-GFP
Performance index (avoidance/total participated)
Induced
Young
Aged
x
Whole life neuronal expression
elavGS>UAS-dsRNA-GFP
Uninduced
Induced
% survival
Age (days post eclosion)

Supplementary Figure 2. Reducing neuronal actin and F-actin stabilization, and not *GFP*, diminishes age-associated F-actin-rich rods in the brain and extends organismal lifespan.
(a) Immunostaining of brains at 63x magnification from young (10-day-old) and aged (45-day-old) *elavGS>UAS-Fhos-RNAi* flies with or without RU486-mediated transgene expression from day 5 onward, showing F-actin (red channel, phalloidin) and nuclear DNA (blue channel, DAPI). Scale bar is 5 µm. Accompanying diagram indicates brain region where imaging was conducted.
(b) Quantification of mean phalloidin fluorescence intensity in brains as shown in (a). n = 4-6 flies per condition, as indicated. *p (d10 vs. d45 uninduced) = 0.0207, * (d45 uninduced vs. d45 induced) p=0.0294; one-way ANOVA, Tukey's multiple comparisons test.
(c) Immunostaining of brains at 63x magnification from young (10-day-old) and aged (45-day-old) *elavGS>UAS-Fhos-RNAi* flies with or without RU486-mediated transgene expression from day 5 onward, showing F-actin (red channel, phalloidin). Scale bar is 5 µm. Accompanying diagram indicates brain region where imaging was conducted.
(d) Quantification of mean phalloidin fluorescence intensity in brains as shown in (a). n = 4-7 flies per condition, as indicated. ns = non-significant; one-way ANOVA, Tukey's multiple comparisons test.
(e) Immunostaining of brains at 20x magnification from 10-day-old *elavGS>UAS-Fhos-RNAi* flies with or without RU486-mediated transgene expression from day 3 onward, showing DILP2 (green channel, anti-DILP2). Scale bar is 20 µm. Accompanying diagram indicates brain region where imaging was conducted.
(f) Quantification of mean DILP2 fluorescence intensity in brains as shown in (e). n = 10-11 flies per condition, as indicated. ns = non-significant; unpaired t-test.
(g) qPCR analysis of *4E-BP* relative to *GAPDH* in fly heads from 10-day-old *elavGS>UAS-*

Fhos-RNAi flies with or without RU486-mediated transgene expression from day 5 onward. n = 6 biological replicates with 5 dissected heads pooled per replicate. ns = non-significant; unpaired t-test.

(h) qPCR analysis of *4E-BP* relative to *GAPDH* in fly thoraces from 10-day-old *elavGS>UAS-Fhos-RNAi* flies with or without RU486-mediated transgene expression from day 5 onward. n = 6 biological replicates with 5 dissected heads pooled per replicate. ns = non-significant; unpaired t-test.

(i) qPCR analysis of *4E-BP* relative to *GAPDH* in fly guts from 10-day-old *elavGS>UAS-Fhos-RNAi* flies with or without RU486-mediated transgene expression from day 5 onward. n = 6 biological replicates with 5 dissected heads pooled per replicate. ns = non-significant; unpaired t-test.

(j) Survival curve of *tub-Gal80ts,elav-Gal4>UAS-dsRNA-GFP* control flies versus of *tub-Gal80ts,elav-Gal4>UAS-Fhos-RNAi* flies with transgene expression from day 5 onward. ***p < 0.0001, log-rank test. n = 289 *tub-Gal80ts,elav-Gal4>UAS-dsRNA-GFP* flies and 282 *tub-Gal80ts,elav-Gal4>UAS-Fhos-RNAi* biologically independent animals.

(k) Survival curve of *GliaGS>UAS-Fhos-RNAi* flies with or without RU486-mediated transgene expression from day 5 onward. ns = non-significant, log-rank test. n = 197 uninduced and 188 induced biologically independent animals.

(l) Survival curve of *elavGS>UAS-Act5c-RNAi* flies with or without RU486-mediated transgene expression from day 30 onward. ***p = <0.0001, log-rank test. n = 197 uninduced and 189 induced biologically independent animals. RU486 or vehicle was provided in the media at a concentration of 25 ug/ml in the indicated treatment group.

(m) Survival curve of *elavGS>UAS-Act42a-RNAi* flies with or without RU486-mediated

transgene expression from day 30 onward. ns = non-significant, log-rank test. n = 204 uninduced and 226 induced biologically independent animals. RU486 or vehicle was provided in the media at a concentration of 50 ug/ml in the indicated treatment group.

(n) Survival curve of *elavGS>UAS-Tsr* flies with or without RU486-mediated transgene expression from day 5 onward. **p = 0.0027, log-rank test. n = 144 biologically independent animals. RU486 or vehicle was provided in the media at a concentration of 50 ug/ml in the indicated treatment group.

(o) Survival curve of *elavGS>UAS-Gelsolin* flies with or without RU486-mediated transgene expression from day 5 onward. ***p = 0.0003, log-rank test. n = 114 uninduced and 134 induced biologically independent animals. RU486 or vehicle was provided in the media at a concentration of 50 ug/ml in the indicated treatment group.

(p) Survival curve of *elavGS>UAS-Act5c-GFP* flies with or without RU486-mediated transgene expression from day 5 onward. ***p = 0.0009, log-rank test. n = 283 uninduced and 297 induced biologically independent animals. RU486 or vehicle was provided in the media at a concentration of 50 ug/ml in the indicated treatment group.

(q) Survival curve of *elavGS>UAS-Act42a-GFP* flies with or without RU486-mediated transgene expression from day 5 onward. *p = 0.0226, log-rank test. n = 252 uninduced and 261 induced biologically independent animals. RU486 or vehicle was provided in the media at a concentration of 50 ug/ml in the indicated treatment group.

(r) Immunostaining brains at 63x magnification from young (10-day-old) and aged (45-day-old) *elavGS>UAS-Act5c-RNAi* flies with or without RU486-mediated transgene expression from day 30 onward, showing F-actin-rich rods (red channel, phalloidin). Scale bar is 5 µm.

(s) Quantification of F-actin-rich rods in brains as shown in (r). n = 6-9 flies per condition, as

indicated. *p = 0.0160, **p = 0.0045, one-way ANOVA, Tukey's multiple comparisons test.

(t) Con-ex feeding assay of 10-day-old *elavGS>UAS-dsRNA-GFP* flies with or without RU486-mediated transgene expression from day 5 onward. n = 6 vials of 10 flies per condition. ns = nonsignificant, unpaired t-test.

(t) Immunostaining brains at 63x magnification from young (10-day-old) and aged (45-day-old) *elavGS>UAS-dsRNA-GFP* flies with or without RU486-mediated transgene expression from day 5 onward, showing F-actin-rich rods (red channel, phalloidin). Scale bar is 5 μm.

(v) Quantification of F-actin-rich rods in brains as shown in (u). n = 4-8 flies per condition, as indicated. *p = 0.0259, **p = 0.0075, one-way ANOVA, Tukey's multiple comparisons test.

(w) Performance index in olfactory aversion training in 37-day-old *elavGS>UAS-dsGFP* flies with or without RU486-mediated transgene expression from day 5 onward, assessed by the number of flies avoiding a shock-associated odor versus the total number of flies participating in the assay. ns = non-significant, *p (young vs aged uninduced) = 0.0294, *p (young vs. aged induced) = 0.0162, one-way ANOVA, Tukey's multiple comparisons test.

(x) Survival curve of *elavGS>UAS-dsRNA-GFP* flies with or without RU486-mediated transgene expression from day 5 onward. ***p = 0.0002, log-rank test. n = 173 RU- and 177 RU+ biologically independent animals. RU486 or vehicle was provided in the media at a concentration of 50 ug/ml in the indicated treatment groups.

Data are presented as scatter plots overlaying mean values +/- SEM.

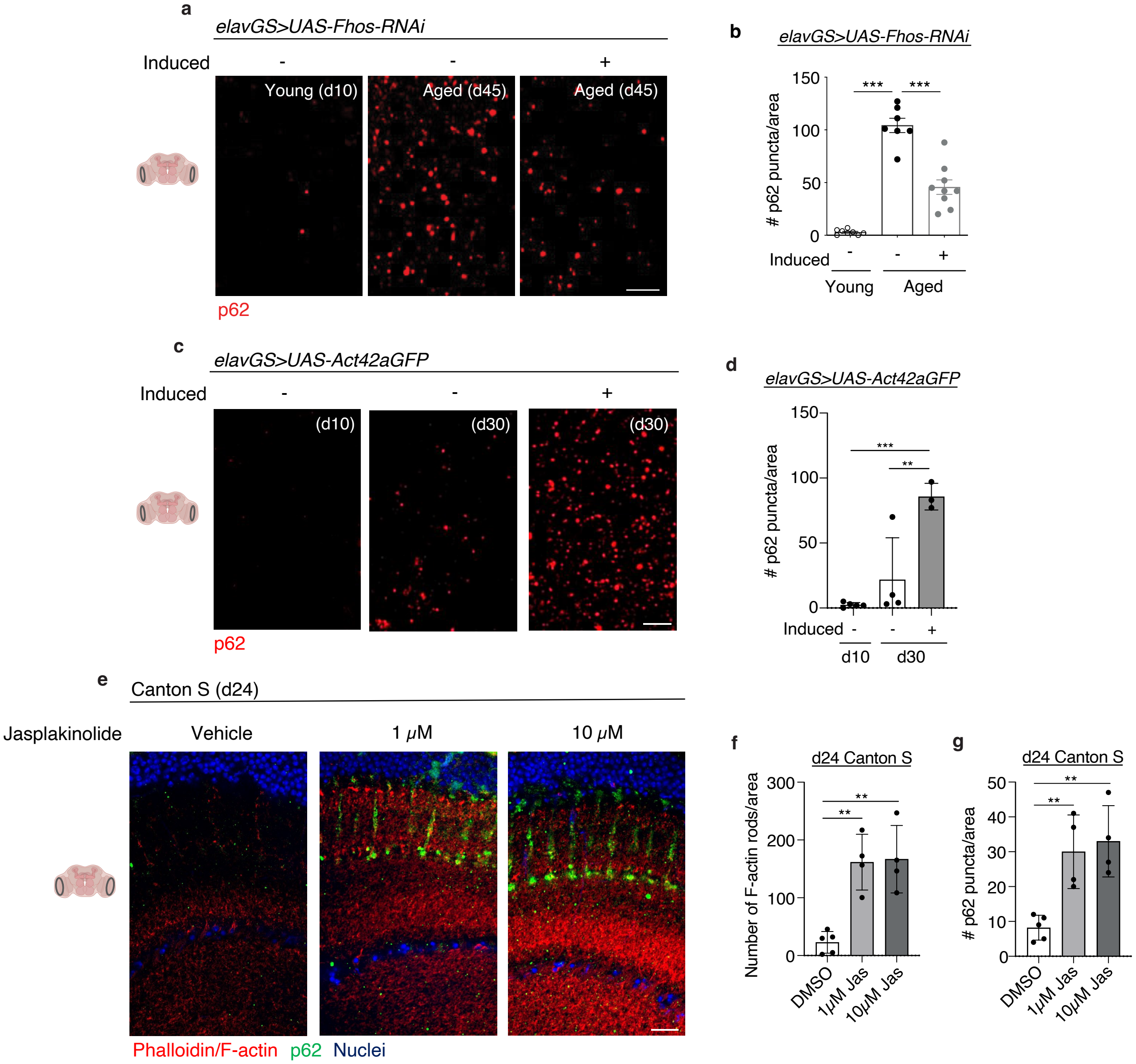

a
elavGS>UAS-Fhos-RNAi
Induced - - +
Young (d10)
Aged (d45)
Aged (d45)
p62
b
elavGS>UAS-Fhos-RNAi
p62 puncta/area
150
100
50
0
***
***
Induced - - +
Young Aged
c
elavGS>UAS-Act42aGFP
Induced - - +
(d10)
(d30)
(d30)
p62
d
elavGS>UAS-Act42aGFP
p62 puncta/area
150
100
50
0
***
**
Induced - - +
d10 d30
e
Canton S (d24)
Jasplakinolide
Vehicle
1 μM
10 μM
Phalloidin/F-actin p62 Nuclei
f
d24 Canton S
Number of F-actin rods/area
300
200
100
0
**
**
DMSO
1μM Jas
10μM Jas
g
d24 Canton S
p62 puncta/area
50
40
30
20
10
0
**
**
DMSO
1μM Jas
10μM Jas

Supplementary Figure 3. F-actin polymerization inversely correlates with autophagic turnover of p62 in the aging *Drosophila* brain.

(a) Immunostaining of brains at 63x magnification from young (10-day-old) and aged (45-day-old) *elavGS>UAS-Fhos-RNAi* flies with or without RU486-mediated transgene induction from day 5 onward, showing p62 accumulation (red channel, anti-Ref(2)P/p62). Scale bar is 5 µm. Accompanying diagram indicates brain region where imaging was conducted.

(b) Quantification of p62 puncta per 2 mm^2 area of brain optic lobes as shown in (a). n = 7-9 biologically independent animals, as indicated. ***$p < 0.0001$ (young vs. aged uninduced), ***$p < 0.0001$ (aged uninduced vs. aged induced); one-way ANOVA, Tukey's multiple comparisons test.

(c) Immunostaining of brains from young (10-day-old) and middle-aged (30-day-old) *elavGS>UAS-Act42a-GFP* flies with or without RU486-mediated transgene induction from day 5 onward, showing p62 accumulation (red channel, anti-Ref(2)P/p62). Scale bar is 5 µm.

(d) Quantification of p62 puncta per 2 mm^2 area of brain optic lobes as shown in (c). n = 3-5 biologically independent animals, as indicated. **$p = 0.0048$ (young vs. aged uninduced), ***$p = 0.0006$ (aged uninduced vs. aged induced); one-way ANOVA, Tukey's multiple comparisons test.

(e) Immunostaining of brains at 63x magnification from 24-day-old Canton S flies fed vehicle (DMSO), 1 µM, or 10 µM jasplakinolide from day 5 onward, showing F-actin (red channel, phalloidin), p62 (green channel, anti-Ref(2)P/p62), and nuclear DNA (blue channel, DAPI). Scale bar is 10 µm.

(f) Quantification of F-actin-rich rods by phalloidin staining per 1 mm^2 area of brain optic lobes as shown in as shown in (e). n = 4-5 biologically independent animals, as indicated. **p =

0.0019 (DMSO vs. 1 μM jasplakinolide), **p = 0.0015 (DMSO vs. 10 μM jasplakinolide); one-way ANOVA, Tukey's multiple comparisons test.

(g) Quantification of p62 puncta per 1 mm^2 area of brain optic lobes as shown in (e). n = 4-5 biologically independent animals, as indicated. **p = 0.0077 (DMSO vs. 1 μM jasplakinolide), **p = 0.0033 (DMSO vs. 10 μM jasplakinolide); one-way ANOVA, Tukey's multiple comparisons test. Data are presented as scatter plots overlaying mean values +/- SEM.

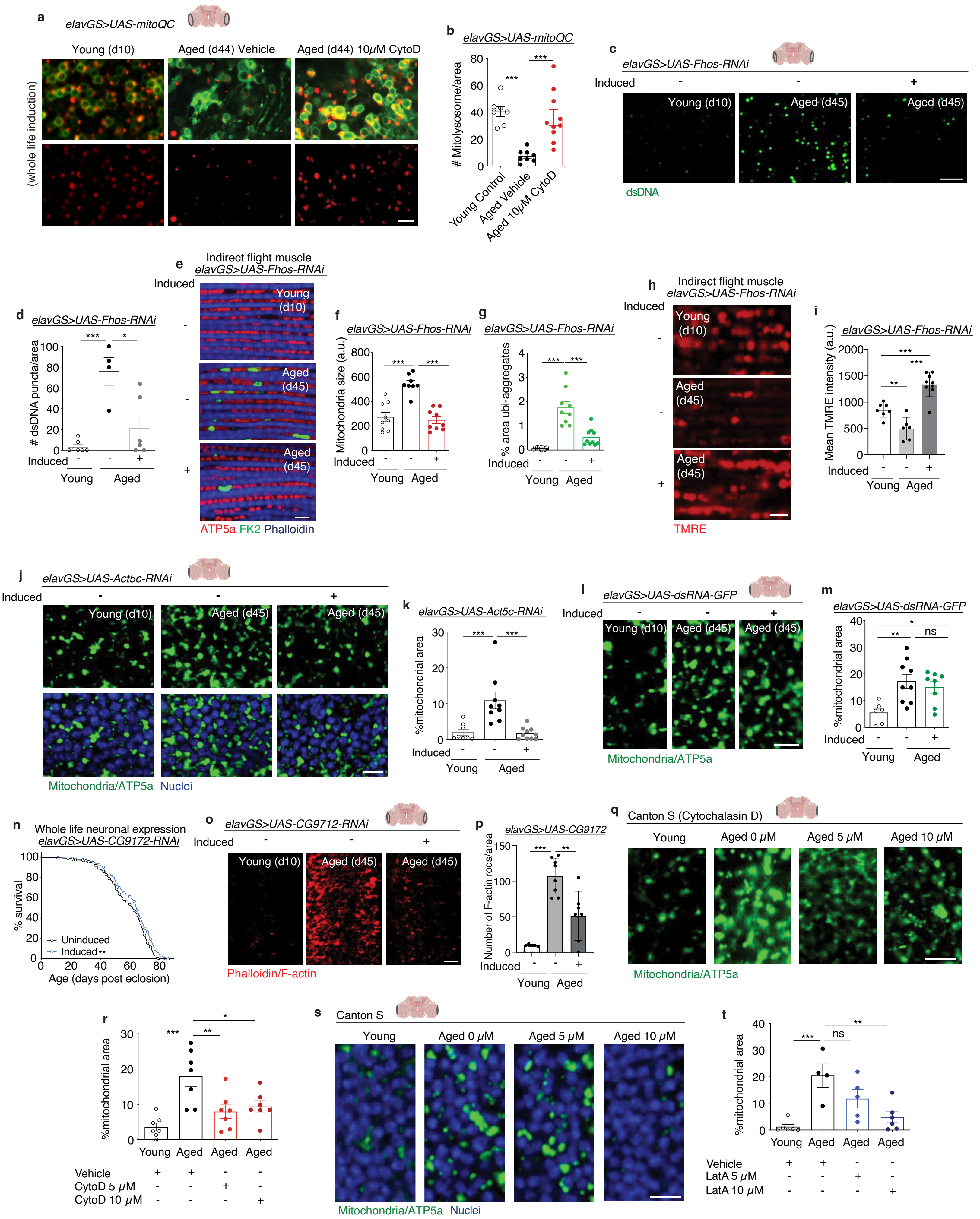
a
elavGS>UAS-mitoQC
Young (d10)
Aged (d44) Vehicle
Aged (d44) 10μM CytoD
(whole life induction)
b
elavGS>UAS-mitoQC
Mitolysosome/area
Young Control
Aged Vehicle
Aged 10μM CytoD
c
elavGS>UAS-Fos-RNAi
Induced
Young (d10)
Aged (d45)
dsDNA
d
elavGS>UAS-Fos-RNAi
dsDNA puncta/area
Induced
Young
Aged
e
Indirect flight muscle
elavGS>UAS-Fos-RNAi
ATP5a FK2 Phalloidin
f
elavGS>UAS-Fos-RNAi
Mitochondria size (a.u.)
g
elavGS>UAS-Fos-RNAi
% area ubi-aggregates
h
Indirect flight muscle
elavGS>UAS-Fos-RNAi
TMRE
i
elavGS>UAS-Fos-RNAi
Mean TMRE intensity (a.u.)
j
elavGS>UAS-Act5c-RNAi
Mitochondria/ATP5a Nuclei
k
elavGS>UAS-Act5c-RNAi
%mitochondrial area
l
elavGS>UAS-dsRNA-GFP
Mitochondria/ATP5a
m
elavGS>UAS-dsRNA-GFP
%mitochondrial area
n
Whole life neuronal expression
elavGS>UAS-CG9172-RNAi
% survival
Uninduced
Induced**
Age (days post eclosion)
o
elavGS>UAS-CG9712-RNAi
Phalloidin/F-actin
p
elavGS>UAS-CG9172
Number of F-actin rods/area
q
Canton S (Cytochalasin D)
Young
Aged 0 μM
Aged 5 μM
Aged 10 μM
Mitochondria/ATP5a
r
%mitochondrial area
Vehicle
CytoD 5 μM
CytoD 10 μM
s
Canton S
Mitochondria/ATP5a Nuclei
t
%mitochondrial area
Vehicle
LatA 5 μM
LatA 10 μM

Supplementary Figure 4. Genetic and pharmacological reduction of F-actin polymerization promotes mitophagy and reduces age-associated mitochondrial accumulation.

(a) Mito-QC of brains at 63x magnification from 10-day-old and 44-day-old flies given vehicle (DMSO) or 10μM cytochalasin D from days 37-44 post eclosion. Images shown of merged GFP and mCherry along with punctate mCherry-only foci (from merged images where GFP has been quenched; mitolysosomes). Scale bar is 5 μm. Accompanying diagrams indicate brain region where imaging was conducted.

(b) Quantification of mitolysosomes per 4 mm^2 brain area as shown in (a). n 7-10 biologically independent animals per condition, as indicated. ***p (young vs. aged vehicle) = 0.0003, ***p (aged vehicle vs. aged 10 μM CytoD) = 0.0005; one-way ANOVA/Tukey's multiple comparisons test.

(c) Immunostaining of brains at 63x magnification of young (10-day-old) and aged (45-day-old) *elavGS>UAS-Fhos-RNAi* flies with or without RU486-mediated transgene induction from day 5 onward, showing non-nuclear double-stranded DNA (green channel, anti-dsDNA). Scale bar is 5 μm.

(d) Quantification of dsDNA puncta in brain as shown in (c). n = 4-7 biologically independent animals, as indicated. **p=0.0038, ***p=0.0002; one-way ANOVA, Tukey's multiple comparisons test.

(e) Immunostaining of indirect flight muscles at 63x magnification of young (10-day-old) and aged (45-day-old) *elavGS>UAS-Fhos-RNAi* flies with or without RU486-mediated transgene induction from day 5 onward, showing mitochondrial morphology (red channel, anti-ATP5a), polyubiquitinated aggregates (green channel, anti-FK2), and F-actin (blue channel, phalloidin). Scale bar is 10 μm.

(f) Quantification of mitochondria size in indirect flight muscles as shown in (e). n = 8-9 biologically independent animals, as indicated. ***p <0.0001; one-way ANOVA, Tukey's multiple comparisons test.

(g) Quantification of % area of polyubiquitinated aggregates in indirect flight muscles as shown in (e). n = 8-10 biologically independent animals, as indicated. ***p <0.0001; one-way ANOVA, Tukey's multiple comparisons test.

(h) Staining of indirect flight muscles at 63x magnification from young (10-day-old) and aged (45-day-old) *elavGS>UAS-Fhos-RNAi* flies with or without RU486-mediated transgene induction from day 5 onward, showing TMRE fluorescence. Scale bar is 5 µm.

(i) Quantification of mitochondrial membrane potential measured by TMRE staining as shown in (h). n = 6-9 biologically independent animals per condition, as indicated. **p (d10 vs. d45 uninduced) = 0.0053, ***p (d10 uninduced vs. d45 induced) = 0.0003, ***p (d45 uninduced vs. d45 induced) < 0.0001; one-way ANOVA/Tukey's multiple comparisons test.

(j) Immunostaining of brains at 63x magnification from young (10-day-old) and aged (45-day-old) *elavGS>UAS-Act5c-RNAi* flies with or without RU486-mediated transgene induction from day 5 onward, showing mitochondrial morphology (green channel, anti-ATP5a) and nuclear DNA (blue channel, stained with To-Pro-3). Scale bar is 5 µm.

(k) Quantification of mitochondrial area in brains as shown in (c). n = 8-10 biologically independent animals per condition, as indicated. ns = non-significant, ***p (young vs. aged uninduced) = 0.0009 ***p (aged uninduced vs. aged induced) = 0.0003; one-way ANOVA/Tukey's multiple comparisons test.

(l) Immunostaining of brains from young (10-day-old) and aged (45-day-old) *elavGS>UAS-dsRNA-GFP* flies with or without RU486-mediated transgene induction from day 5 onward,

showing mitochondrial morphology (green channel, anti-ATP5a). Scale bar is 5 μm.

(m) Quantification of mitochondrial area in brains as shown in (l). n = 6-9 biologically independent animals per condition, as indicated. ns = non-significant, *p = 0.0371, **p = 0.0077; one-way ANOVA/Tukey's multiple comparisons test.

(n) Survival curve of *elavGS>UAS-CG9172-RNAi* flies with or without RU486-mediated transgene expression from day 5 onward. **p = 0.0014, log-rank test. n = 283 uninduced and 271 induced biologically independent animals.

(o) Immunostaining of brains at 63x magnification from young (10-day-old) and aged (45-day-old) *elavGS>UAS-CG9172-RNAi* flies with or without RU486-mediated transgene expression from day 5 onward, showing F-actin-rich rods (red channel, phalloidin). Scale bar is 5 μm.

(p) Quantification of F-actin-rich rods by phalloidin staining per 1 mm^2 area of brain optic lobes as shown in (o). n = 5-8 flies per condition, as indicated. **p = 0.0019, ***p < 0.0001, one-way ANOVA, Tukey's multiple comparisons test.

(q) Immunostaining of brains from young (10-day-old) and aged (44-day-old) Canton S flies given vehicle (DMSO), 5 μM, or 10μM cytochalasin D as indicated from days 37-44 post eclosion, showing mitochondrial morphology (green channel, anti-ATP5a). Scale bar is 5 μm.

(r) Quantification of mitochondrial area in brains as shown in (e). n = 7 biologically independent animals per condition. *p = 0.0277, **p = 0.0083, ***p = 0.0002; one-way ANOVA/Tukey's multiple comparisons test.

(s) Immunostaining of brains from young (10-day-old) and aged (45-day-old) Canton S flies given vehicle (DMSO), 5 μM, or 10μM Latrunculin A as indicated from days 37-44 post eclosion, showing mitochondrial morphology (green channel, anti-ATP5a) and nuclear DNA (blue channel, stained with To-Pro-3). Scale bar is 5 μm.

(t) Quantification of mitochondrial area in brains as shown in (s). n = 4-7 biologically independent animals per condition, as indicated. p** = 0.0046, ***p = 0.0007, ns = non-significant; one-way ANOVA/Tukey's multiple comparisons test. Data are presented as scatter plots overlaying mean values +/- SEM.

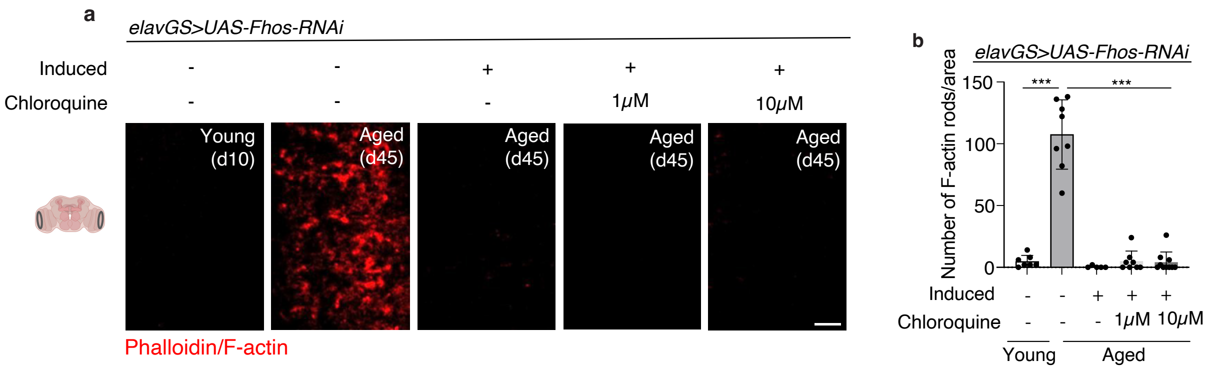
a
elavGS>UAS-Fhos-RNAi
Induced - - + + +
Chloroquine - - - 1μM 10μM
Young (d10)
Aged (d45)
Aged (d45)
Aged (d45)
Aged (d45)
Phalloidin/F-actin
b
elavGS>UAS-Fhos-RNAi
Number of F-actin rods/area
150
100
50
0
***
***
Induced - - + + +
Chloroquine - - - 1μM 10μM
Young
Aged

Supplementary Figure 5. Neuronal knockdown of *Fhos* reduces age-associated F-actin-rich rods in the brains of *Drosophila* treated with a pharmacological inhibitor of autophagy.

(a) Immunostaining of brains at 63x magnification from young (10-day-old) and aged (45-day-old) *elavGS>UAS-Fhos-RNAi* flies with or without RU486-mediated transgene expression from day 5 onward and treated with vehicle (water), 1 μM chloroquine, or 10 μM chloroquine, as indicated. Images show F-actin (red channel, phalloidin). Scale bar is 5 μm. Accompanying diagram indicates brain region where imaging was conducted.

(b) Quantification of F-actin-rich rods per 1 mm^2 area of brain optic lobes as shown in (a). n = 5-13 flies per condition, as indicated. ***$p < 0.0001$; one-way ANOVA, Tukey's multiple comparisons test. Data are presented as scatter plots overlaying mean values +/- SEM.
