# Supplementary material for: Accumulation of F-actin drives brain aging and limits healthspan in Drosophila
Source: Nat Commun. 2024 Oct 25;15:9238. doi: 10.1038/s41467-024-53389-w (PMC11512044; doi:10.1038/s41467-024-53389-w)
Supplement: Supplementary file 3 — Reporting Summary [file 41467_2024_53389_MOESM3_ESM.pdf]

nature portfolio

Corresponding author(s): David W. Walker

Last updated by author(s): Sep 5, 2024

Reporting Summary

Nature Portfolio wishes to improve the reproducibility of the work that we publish. This form provides structure for consistency and transparency in reporting. For further information on Nature Portfolio policies, see our Editorial Policies and the Editorial Policy Checklist.

Statistics

For all statistical analyses, confirm that the following items are present in the figure legend, table legend, main text, or Methods section.

n/a	Confirmed	
☐	☒	The exact sample size (*n*) for each experimental group/condition, given as a discrete number and unit of measurement
☐	☒	A statement on whether measurements were taken from distinct samples or whether the same sample was measured repeatedly
☐	☒	The statistical test(s) used AND whether they are one- or two-sided<br>*Only common tests should be described solely by name; describe more complex techniques in the Methods section.*
☐	☒	A description of all covariates tested
☐	☒	A description of any assumptions or corrections, such as tests of normality and adjustment for multiple comparisons
☐	☒	A full description of the statistical parameters including central tendency (e.g. means) or other basic estimates (e.g. regression coefficient) AND variation (e.g. standard deviation) or associated estimates of uncertainty (e.g. confidence intervals)
☐	☒	For null hypothesis testing, the test statistic (e.g. *F*, *t*, *r*) with confidence intervals, effect sizes, degrees of freedom and *P* value noted<br>*Give P values as exact values whenever suitable.*
☒	☐	For Bayesian analysis, information on the choice of priors and Markov chain Monte Carlo settings
☒	☐	For hierarchical and complex designs, identification of the appropriate level for tests and full reporting of outcomes
☒	☐	Estimates of effect sizes (e.g. Cohen's *d*, Pearson's *r*), indicating how they were calculated

Our web collection on statistics for biologists contains articles on many of the points above.

Software and code

Policy information about availability of computer code

Data collection	Fluorescent image acquisition: Zeiss ZEN software (version 2.6, Carl Zeiss)<br>qRT-PCR: BioRad CFX manager version 3.1 (Bio-Rad Laboratories)<br>Target number and size: ImageJ (version 1.53c)
Data analysis	Excel 2016 and GraphPad Prism 10 were used for general data analysis.<br>Images were analyzed using ImageJ 1.53f.<br>Additional details are provided in the materials and methods section.

For manuscripts utilizing custom algorithms or software that are central to the research but not yet described in published literature, software must be made available to editors and reviewers. We strongly encourage code deposition in a community repository (e.g. GitHub). See the Nature Portfolio guidelines for submitting code & software for further information.

Data

Policy information about availability of data

All manuscripts must include a data availability statement. This statement should provide the following information, where applicable:

- Accession codes, unique identifiers, or web links for publicly available datasets
- A description of any restrictions on data availability
- For clinical datasets or third party data, please ensure that the statement adheres to our policy

All data generated or analyzed during this study are included in the figures and text with representative images accompanying quantified results where applicable unless otherwise noted. Further information is available from the corresponding author upon reasonable request.

Research involving human participants, their data, or biological material

Policy information about studies with human participants or human data. See also policy information about sex, gender (identity/presentation), and sexual orientation and race, ethnicity and racism.

Reporting on sex and gender	*Use the terms sex (biological attribute) and gender (shaped by social and cultural circumstances) carefully in order to avoid confusing both terms. Indicate if findings apply to only one sex or gender; describe whether sex and gender were considered in study design; whether sex and/or gender was determined based on self-reporting or assigned and methods used. Provide in the source data disaggregated sex and gender data, where this information has been collected, and if consent has been obtained for sharing of individual-level data; provide overall numbers in this Reporting Summary. Please state if this information has not been collected. Report sex- and gender-based analyses where performed, justify reasons for lack of sex- and gender-based analysis.*
Reporting on race, ethnicity, or other socially relevant groupings	*Please specify the socially constructed or socially relevant categorization variable(s) used in your manuscript and explain why they were used. Please note that such variables should not be used as proxies for other socially constructed/relevant variables (for example, race or ethnicity should not be used as a proxy for socioeconomic status). Provide clear definitions of the relevant terms used, how they were provided (by the participants/respondents, the researchers, or third parties), and the method(s) used to classify people into the different categories (e.g. self-report, census or administrative data, social media data, etc.) Please provide details about how you controlled for confounding variables in your analyses.*
Population characteristics	*Describe the covariate-relevant population characteristics of the human research participants (e.g. age, genotypic information, past and current diagnosis and treatment categories). If you filled out the behavioural & social sciences study design questions and have nothing to add here, write "See above."*
Recruitment	*Describe how participants were recruited. Outline any potential self-selection bias or other biases that may be present and how these are likely to impact results.*
Ethics oversight	*Identify the organization(s) that approved the study protocol.*

Note that full information on the approval of the study protocol must also be provided in the manuscript.

Field-specific reporting

Please select the one below that is the best fit for your research. If you are not sure, read the appropriate sections before making your selection.

☒ Life sciences ☐ Behavioural & social sciences ☐ Ecological, evolutionary & environmental sciences

For a reference copy of the document with all sections, see nature.com/documents/nr-reporting-summary-flat.pdf

Life sciences study design

All studies must disclose on these points even when the disclosure is negative.

Sample size	No statistical methods were used to pre-determine sample sizes. Sample sizes used in this study are similar to those reported in previous publications (Ulgherait et al., 2014, Cell Reports; Rana et al., 2017, Nat Commun; Aparicio et al., 2019, Cell Reports).
Data exclusions	No data were excluded from the analyses.
Replication	All data presented were from independent biological replicates or independent experiments. All attempts at replication validated results. When lifespan extensions were observed, results were confirmed in at least one independent experiment.
Randomization	Experimental and control flies were maintained under the same conditions and allocated to treatments/group randomly. Steps were taken to avoid batch effects.
Blinding	Blinding was not always possible during experimental setup given that investigators needed to carefully document the genotypes of flies when generating crosses or to track assigned groups being maintained on RU vs. vehicle throughout lifespans. Blinding was performed when possible, specifically when conducting microscopy for TMRE, Atg8a-tandem, and mitoQC. All experiments were conducted under the same

conditions, and control and experimental samples were treated equally and in parallel to exclude bias. Additionally, all images were taken in the same location and depth in each tissue type.

Reporting for specific materials, systems and methods

We require information from authors about some types of materials, experimental systems and methods used in many studies. Here, indicate whether each material, system or method listed is relevant to your study. If you are not sure if a list item applies to your research, read the appropriate section before selecting a response.

Materials & experimental systems

n/a	Involved in the study
☐	☒ Antibodies
☒	☐ Eukaryotic cell lines
☒	☐ Palaeontology and archaeology
☐	☒ Animals and other organisms
☒	☐ Clinical data
☒	☐ Dual use research of concern
☒	☐ Plants

Methods

n/a	Involved in the study
☒	☐ ChIP-seq
☒	☐ Flow cytometry
☒	☐ MRI-based neuroimaging

Antibodies

Antibodies used

mouse anti-ATP5a (15H4C4, abcam)
mouse anti-actin (JLA20, DSHB)
mouse anti-FK2 (BML-PW8810-0500, ENZO)
rabbit anti-atg8a (home made, Rana et al., 2017)
rabbit-anti-ref(2)P (home made Rana et al., 2017)
mouse anti-dsDNA (ab27156, abcam)
rabbit-anti-DILP2 (a generous gift from Dr. Seung Kim),
mouse-anti-neuroglian (BP 104, DSHB)
rat-anti-N-cadherin (DN-Ex #8, DSHB)
rabbit or mouse AlexaFluor-488 (A-11001 or A-11008, Thermo Fisher Scientific)
rabbit, mouse, or rat AlexaFluor-568 (A-11031, A-11036, or A-11077, Thermo Fisher Scientific)
phalloidin AlexaFluor-568 or AlexaFluor-488 (A12380, or A12379 Thermo Fisher Scientific)

Validation

All antibodies were used in accordance to the manufacturer guidelines.

mouse anti-ATP5a (15H4C4, abcam) was validated by the manufacturer and in the following publications:
Bawa S et al.,Elife (2020) 9:e52358
Chen PL et al., Nat Commun (2020) 11(1):2592
Aparicio R et al., Cell Rep (2019) 28(4):1029-1040.e5

rabbit anti-actin (JLA20, DSHB) was validated by the manufacturer and the following publications:
Frappaoloa A et al., Cell Death Dis (2022) 27;13(11):1003
Gordon D et al., PLoS Genet (2017) 2;13(4):e1006698
Ordonez D et al., Neuron (2018) 97(1): 108–124.e6

mouse anti-FK2 (BML-PW8810-0500, ENZO) was validated by the manufacturer and the following publications:
Tamai K et al., Am J Pathol (2008) 173(6):1806-17
Aparicio R et al., Cell Rep (2019) 28(4):1029-1040.e5

rabbit anti-ref(2)P (home made, Rana et al., 2017) was validated by the following publications:
Rana A et al., Nat Commun (2017) 8(1):448
Aparicio R et al., Cell Rep (2019) 28(4):1029-1040.e5

rabbit anti-atg8a (home made, Rana et al., 2017) was validated by the following publications:
Rana A et al., Nat Commun (2017) 8(1):448
Aparicio R et al., Cell Rep (2019) 28(4):1029-1040.e5

mouse anti-dsDNA (ab27156, abcam) was validated by the manufacturer and the following publications:
Hu Q et al., Cell Rep (2020) 30:1235-1245.e4
Aparicio R et al., Cell Rep (2019) 28(4):1029-1040.e5

rabbit anti-DILP2 (a generous gift from Dr. Seung Kim) was validated by the manufacturer and the following publications:
Ulgherait M et al., Cell Rep (2014) 8(6): 1767–1780
Park S et al., PLoS Genet (2014) 10(8): e1004555

mouse-anti-neuroglian (BP 104, DSHB) was validated by the manufacturer and the following publications:
Croteau-Chonka E et al., ELife (2022) 28:11:e70015
Shepherd D et al, J Comp Neurol (2016) 1;524(13):2677-95

rat-anti-N-cadherin (DN-Ex #8, DSHB) was validated by the manufacturer and the following publications:
Sampson M et al., PLoS Genet (2020) 16(8):e1009003
Contreras E et al., Sci Rep (2018) 8(1):13332

rabbit or mouse AlexaFluor-488 (A-11001 or A-11008, Thermo Fisher Scientific) were validated by the manufacturer and the following publications:
Yun HY et al., PLoS Biol (2019) 17(7):e3000367
Foggetti A et al., Cell Rep (2019) 27(13):3725-3732.e5

rabbit, mouse, or rat AlexaFluor-568 (A-11031, A-11036, or A-11077, Thermo Fisher Scientific) were validated by the manufacturer and the following publications:
Schips TG et al., Nat Commun (2019) 10(1):76
Namiki S et al., Elife (2018) 7:e34272
Sapmaz A et al., Nat Commun (2019) 10(1):1454.
Yip SH et al., Cell Rep (2019) 26(7):1787-1799.e5

phalloidin AlexaFluor-568 or AlexaFluor-488 (A12380, or A12379 Thermo Fisher Scientific) were validated by the manufacturer and the following publications:
Helfand BT et al., J Cell Biol (2002) 157:795-806
Meary F et al., J Biol Chem (2007) 282:14226-14237
Rana A et al., Nat Commun (2017) 8(1):448

Animals and other research organisms

Policy information about studies involving animals; ARRIVE guidelines recommended for reporting animal research, and Sex and Gender in Research

Laboratory animals	Drosophila melanogaster. Female flies were used in experiments and maintained throughout their entire lifespan unless samples were collected at the days specified in corresponding figure legends, specifically on days 10, 30, or 44/45 post eclosion. Crosses generated and used in this study were elavGS>UAS-Fhos-RNAi; elavGS>UAS-Act5c-RNAi; elavGS>UAS-Act42a-RNAi; elavGS>UAS-Tsr; elavGS>UAS-Gelsolin; elavGS>UAS-Act5cGFP; elavGS>UAS-Act42a-GFP; elavGS>GFP-mCherry-ATG8a,UAS-Fhos-RNAi; elavGS>UAS-mito-QC,UAS-lacZ; elavGS>UAS-mito-QC; elavGS>UAS-mito-QC,UAS-Fhos-RNAi; elavGS>UAS-Atg1RNAi,UAS-Fhos-RNAi; elavGS>UAS-dsRNA-GFP; tub-Gal80ts,elav-Gal4>UAS-Fhos-RNAi; and tub-Gal80ts,elav-Gal4>UAS-dsRNA-GFP
Wild animals	Our study did not involve any wild animals.
Reporting on sex	*Indicate if findings apply to only one sex; describe whether sex was considered in study design, methods used for assigning sex. Provide data disaggregated for sex where this information has been collected in the source data as appropriate; provide overall numbers in this Reporting Summary. Please state if this information has not been collected. Report sex-based analyses where performed, justify reasons for lack of sex-based analysis.*
Field-collected samples	Our study did not involve any field-collected samples.
Ethics oversight	No ethical approval was required.

Note that full information on the approval of the study protocol must also be provided in the manuscript.

Plants

Seed stocks	*Report on the source of all seed stocks or other plant material used. If applicable, state the seed stock centre and catalogue number. If plant specimens were collected from the field, describe the collection location, date and sampling procedures.*
Novel plant genotypes	*Describe the methods by which all novel plant genotypes were produced. This includes those generated by transgenic approaches, gene editing, chemical/radiation-based mutagenesis and hybridization. For transgenic lines, describe the transformation method, the number of independent lines analyzed and the generation upon which experiments were performed. For gene-edited lines, describe the editor used, the endogenous sequence targeted for editing, the targeting guide RNA sequence (if applicable) and how the editor was applied.*
Authentication	*Describe any authentication procedures for each seed stock used or novel genotype generated. Describe any experiments used to assess the effect of a mutation and, where applicable, how potential secondary effects (e.g. second site T-DNA insertions, mosiacism, off-target gene editing) were examined.*
